# Standardized Instance‐Level Quantification of CD34‐Positive Vessels in Lymph Node Whole‐Slide Images Using U‐Net

**DOI:** 10.1111/pin.70125

**Published:** 2026-05-11

**Authors:** Satomi Omotani, Natsumi Yonemoto, Akifumi Muramoto, Motohiro Kobayashi

**Affiliations:** ^1^ Department of Pathology, Faculty of Medical Sciences University of Fukui Eiheiji Fukui Japan

**Keywords:** AI, CD34, deep learning, lymph nodes, vessels

## Abstract

Deep learning enhances quantitative analysis of blood vessels in whole‐slide images (WSIs), leading to precise and reliable counting. However, counting vessels in lymph nodes—which contain distinctive high endothelial venules (HEVs)—has not been systematically evaluated. Artificial intelligence (AI) approaches to vessel‐counting fall into two categories: semantic segmentation, which provides a continuous mask, and instance segmentation, which treats each vessel as a separate object; prior work has emphasized the former. To address this gap, we adopted an instance‐level approach to detect and count individual vessels. We developed a convolutional neural network (CNN) to quantify CD34‐positive vessels in lymph‐node WSIs, using U‐Net, a widely used biomedical segmentation architecture. The model was trained over 4.0 million (4.0 M) iterations, followed by a fixed post‐processing procedure to extract vessel instances. At 3.0 M, the model most faithfully captured CD34‐positive vascular morphology. In the validation set, this checkpoint showed the highest morphological similarity. Furthermore, detection performance was robust with minimal variation across lymph nodes and training checkpoints. Using a simple fixed procedure, our method achieves accurate vessel detection and counting in lymph‐node WSIs. This model provides a practical basis for standardized quantification and for studying lymph‐node vascular morphology across normal, inflammatory, and lymphomatous states.

## Graphical abstract

We develop a deep learning approach to detect and count individual CD34‐positive vessels in lymph‐node whole‐slide images. Using an object‐level method based on U‐Net, the model captures vascular morphology, and vessel counts are corrected through simple fixed post‐processing. This procedure provides a practical basis for standardized vascular quantification in digital pathology and for broader analytical and research applications.

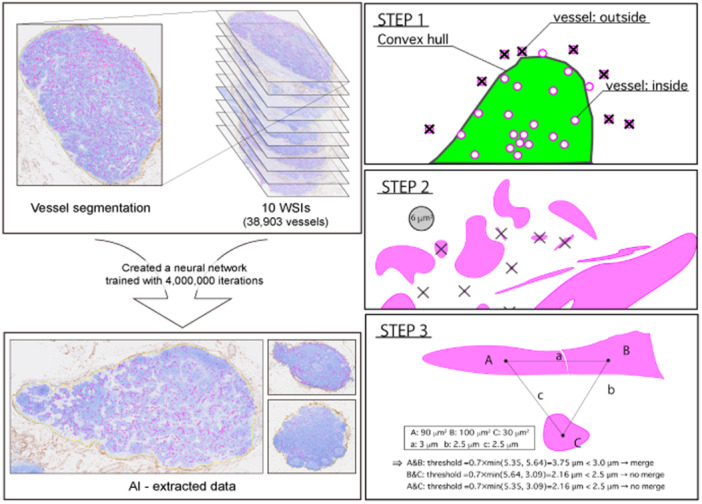

**Abbreviations**
AI: artificial intelligence
CNN: convolutional neural network
FN: false negative
FP: false positive
H&E: hematoxylin and eosin
ICC: intraclass correlation coefficient
IoU: intersection over union
k: kilo
M: million
MAE: mean absolute error
NMS: non‐maximum suppression
ROI: region of interest
TN: true negative
TP: true positive
WSI: whole‐slide image

## 1 Introduction

Blood vessels play essential roles in tissue homeostasis, immune surveillance, and tumor progression. High endothelial venules (HEVs) and HEV‐like vessels serve as specialized portals for lymphocyte trafficking and reflect immune activation in reactive and neoplastic conditions [1, 2, 3, 4]. Quantitative analysis of these vessels can reveal the degree of immune response, tissue remodeling, and disease progression.

Before the era of whole‐slide imaging, vascular evaluation relied on manual counting within “representative” microscopic fields. Because the exact same field could not be revisited, this approach showed poor interobserver agreement and imposed substantial observer workload. Digital pathology now enables quantitative, repeatable assessment of entire tissue sections. Large validation studies have confirmed that whole‐slide imaging is suitable for primary diagnosis in routine pathology [5, 6, 7]. In parallel, machine learning—particularly convolutional neural networks (CNNs)—has advanced rapidly, achieving accurate segmentation and object detection in histopathology images [6, 8]. CNNs are deep learning models that learn spatial patterns in images. CNNs emerged in the late 1980s with LeNet, an early model for handwritten digit recognition [9]. After the 2012 ImageNet breakthrough, CNNs became a foundation of modern image analysis, including biomedical segmentation and detection with architectures such as U‐Net [8, 10, 11, 12]. These advances have accelerated applications in pathology and medical imaging [8]. Deep‐learning‐based vascular analysis has been applied in various settings, including glioma microvascularity and hematoxylin and eosin (H&E)‐based microvessel prediction, supporting automated vessel quantification as a practical approach [13, 14, 15].

Nevertheless, many artificial intelligence (AI)‐based pathology studies emphasize pixel‐level metrics and patch‐based evaluations, whereas instance‐level vessel detection and counting with standardized post‐processing have been evaluated far less often [15, 16]. Semantic segmentation assigns a class to every pixel to produce a continuous mask (e.g., a tumor region), whereas instance segmentation detects and separates individual vessels as separate objects, enabling counting and per‐object measurements. To support both vessel counting and morphological assessment, we adopted an instance‐level approach. Pixel‐level metrics such as the Dice coefficient and intersection over union (IoU) quantify overlap between predicted and reference regions, whereas instance‐level metrics (precision, recall, F1) evaluate whether each vessel is correctly identified as a separate object [15, 17]. These object‐based measures align with practical pathology tasks that require accurate identification and enumeration of biologic structures such as nuclei, glands, and vessels.

To our knowledge, instance‐level agreement in lymph‐node vessel segmentation has not been systematically evaluated. We therefore developed and validated an AI‐based procedure using U‐Net [10, 16, 17], trained up to 4.0 million iterations (4.0 M) with a fixed post‐processing procedure. Our aim was to standardize the quantitative assessment of CD34‐positive vessels in lymph nodes and to compare AI with expert annotations at both object and pixel levels, with instance‐level performance as the primary endpoint.

## 2 Materials and Methods

### 2.1 Study Design and Setting

We conducted a single‐center, retrospective validation study at the Faculty of Medical Sciences, University of Fukui.

### 2.2 Slide Preparation and Whole‐Slide Images

**Figure 1:**
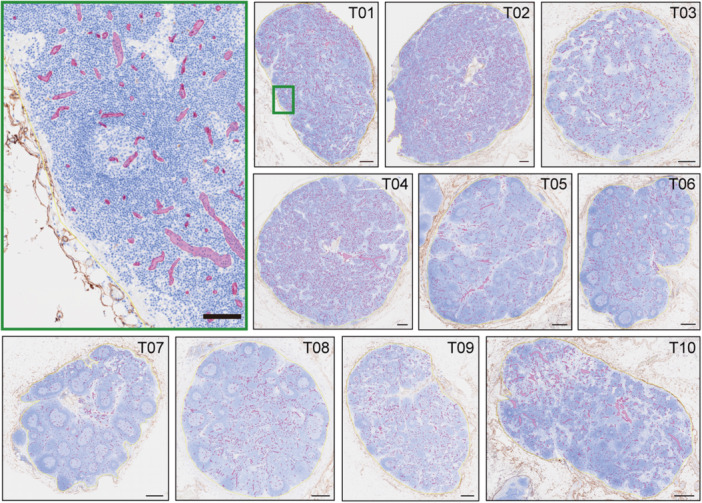
Overview of CD34‐immunostained lymph‐node dataset used for model training and validation. Representative WSIs from the training set (T01–T10) illustrating variation in nodal morphology and vascular density. Magenta overlays indicate model‐predicted vessel regions used for annotation and model refinement. Image in the upper‐left green frame: the area indicated by the green box in T01 is magnified. Scale bars = 500 µm on T01‐10, = 100 µm on green‐framed image. WSI, whole‐slide image.

We used formalin‐fixed, paraffin‐embedded tissue blocks that contained multiple anonymized lymph nodes from cancer‐surgery patients without nodal metastasis (1988–2025). We stained sections for CD34 (clone QBEND10, mouse IgG; Immunotech, Luminy, France), used Histofine Simple Stain MAX‐PO MULTI (Nichirei, Tokyo, Japan) as the secondary reagent, developed the chromogenic signal with 3,3′‐diaminobenzidine (DAB), and added a hematoxylin counterstain. Slides were scanned at 40× as whole‐slide images (WSIs) on the SLIDEVIEW VS200 (Evident, Tokyo, Japan) with a pixel size of 0.137 µm per pixel. Individual lymph nodes (T01–T10 and L01–L03) were cropped from each WSI. The validation cases (L01–L03) were selected to represent diversity in staining intensity and lymph node size. Two cases came from patients independent of the training set, while the third represented a distinct lymph node from a training‐set patient. Image overlap between the training and validation sets was strictly avoided, ensuring morphological diversity sufficient for rigorous performance evaluation. The nodal region was then annotated as a polygonal region of interest (ROI) bounded by the capsule; the hilum and perinodal adipose tissue were excluded (Figure 1).

### 2.3 Reference Standard

**Figure 2:**
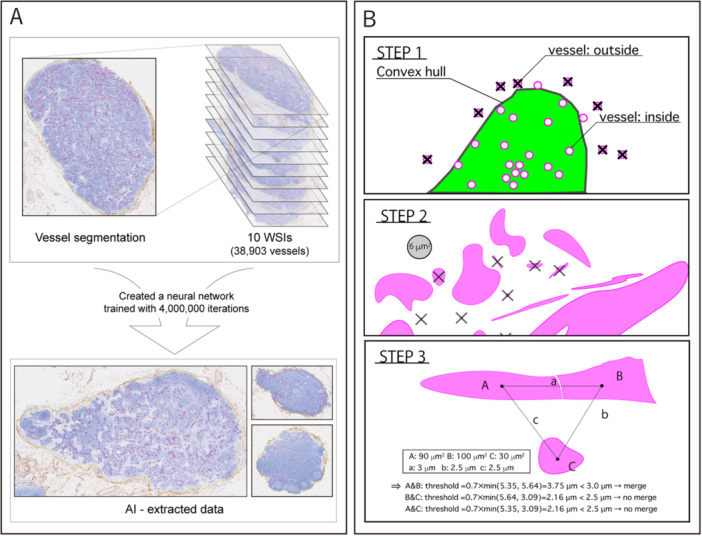
Data, model, and post‐processing procedure. (A) Overview of training and inference using CD34‐immunostained WSIs. A U‐Net backbone was trained up to 4.0 million iterations; representative detections on lymph‐node WSIs are shown. (B) Fixed post‐processing applied to all tests: removal of predictions outside the capsule using a convex‐hull proxy ROI (Step 1), exclusion of small fragments (< 6 µm²) (Step 2), and NMS = 0.7 (Step 3). NMS, non‐maximum suppression; ROI, region of interest; WSI, whole‐slide image.

We established reference‐standard vessel masks through a staged annotation process (Figure 2A). A medical student (S.O.) generated initial annotations with a provisional AI model; a pathologist (N.Y.) corrected them, and two other pathologists (A.M., M.K.) independently reviewed all annotations, blinded to the AI outputs. Weakly CD34‐positive vessels were included when morphologically consistent, and tiny objects (< 10 pixels in area, ≈0.19 µm²) were excluded. Adjacent cross‐sections judged to represent a single tortuous vessel were merged. Discrepancies were resolved by consensus, following prior HEV literature [1, 2, 3, 4].

### 2.4 AI Engine and Training

The U‐Net architecture (expert‐channel mode, 25% scale) was configured in the TruAI module of cellSens Dimensions (Evident). A provisional model was trained on approximately 10,000 manually segmented CD34‐positive vessels from three lymph nodes, applied to 10 additional lymph nodes, and then corrected to generate the final masks. The final model was trained on these 10 lymph nodes for up to 4.0 M, with checkpoints at 25 k and every 500 k from 1.0 M through 4.0 M. The input images were derived from WSIs scanned at ×40 magnification. The data size of each Whole Slide Image (WSI) is provided in Table 1.

**Table 1:** ROI‐level morphometry and vessel statistics.

	Vessel count	Area of ROI (×10⁶ μm²)	Perimeter of ROI (μm)	Median of vessel area (min–max) (μm²)	Median of vessel perimeter (min–max) (μm)	Data Size (×10^6^ pixel)
T01	3911	20.05	22661	114.93 (10.94–14 680.03)	48.54 (12.21–1213.05)	1634.95
T02	11421	62.12	39695	115.03 (6.34–31 164.79)	49.89 (11.44–1860.95)	4592.27
T03	2045	15.53	17928	127.74 (9.82–7349.25)	51.46 (11.83–998.78)	1217.65
T04	8766	47.06	31121	92.44 (7.16–155161.40)	44.21 (9.61–5365.51)	3522.43
T05	2001	18.97	19394	105.28 (11.73–22047.96)	47.45 (12.62–1466.51)	1520.98
T06	2483	16.55	20738	90.39 (7.89–4874.89)	44.30 (10.23–1057.35)	1315.60
T07	1649	12.38	20587	63.07 (7.59–2384.72)	33.99 (9.81–369.04)	1302.51
T08	1931	14.31	16810	83.86 (8.15–5541.63)	41.75 (10.41–914.39)	1127.85
T09	2640	20.49	21450	71.36 (6.71–6114.43)	37.64 (9.19–864.82)	1874.21
T10	2056	14.09	19443	99.82 (7.02–26 030.68)	44.18 (10.96–1673.30)	1283.11
L01	1698	12.65	20124	103.01 (15.28–5896.80)	47.33 (15.34–623.04)	1215.50
L02	1511	8.96	17009	107.02 (9.91–7941.98)	48.91 (13.12–1125.42)	1211.47
L03	447	3.48	8723	75.86 (9.50–1176.48)	41.19 (13.89–472.94)	467.47

*Note:* For each ROI: total area, perimeter, vessel count, and median vessel size metrics on CD34‐immunostained lymph‐node WSIs. Abbreviations: LN, lymph node; ROI, region of interest.

The hyperparameters used in TruAI are summarized below. It should be noted that the batch size and learning rate could not be confirmed, as the developer was unable to provide this information upon inquiry—Architecture: U‐Net, Optimizer: Adam, Loss Function: Softmax cross‐entropy, Data Sampling: Balanced sampler (to address class distribution), Normalization: Local contrast normalization, Data Augmentation: 90° rotations and mirroring (no additional image processing augmentation was used).

Segmentation accuracy was evaluated using the Dice coefficient and IoU. The Dice coefficient measures overlap between prediction and ground truth as

$$\mathrm{Dice}=2\mathrm{TP}/\left(2\mathrm{TP}+\mathrm{FP}+\mathrm{FN}\right),$$

while IoU is defined as

$$\mathrm{IoU}=\mathrm{TP}/\left(\mathrm{TP}+\mathrm{FP}+\mathrm{FN}\right).$$

These metrics quantify pixel‐level similarity between predicted and reference vessel masks; higher values indicate better segmentation performance.

### 2.5 Fixed Post‐Processing Procedure

We used a fixed post‐processing procedure to ensure consistent vessel selection and counting (Figure 2B).

1. Because of a software limitation that prevented direct export of ROI coordinates, we used a convex hull (“rubber band”) around all annotation points as a proxy ROI.
2. We excluded objects smaller than 6 µm², which typically represented staining noise. This threshold was selected after a 2–8 µm² sweep that minimized mean absolute error (MAE) with minimal loss of F1.
3. Non‐maximum suppression (NMS) with a distance factor of 0.7 was applied to merge overlapping predictions and retain the largest object [18].

### 2.6 Instance‐Level Matching and Metrics

Predicted and reference vessels were matched in a one‐to‐one manner using the Hungarian method [19]. Predicted and reference vessels were paired when the following conditions were simultaneously satisfied:

Centroid distance:

d(p,g)<min(rp,rg).

Equivalent radius:

$$r=\sqrt{A/\pi }.$$

Relative area difference:

$$|A_{p}-A_{g}|/\mathrm{Ag}<0.30.$$

Here, p and g denote the predicted and ground truth (reference) vessel objects, respectively. d(p,g)is the Euclidean distance between their centroids, r is the equivalent radius of the object, and A is the area. The threshold 0.30 is a dimensionless fraction representing the maximum allowable relative area difference. All matches were assigned in a one‐to‐one manner using the Hungarian algorithm. A reference analysis was also performed using an equivalent‐circle intersection‐over‐union (IoU ≈ 0.50), corresponding to typical Dice/Jaccard thresholds for comparable overlap. We accepted a match when centroid proximity and relative area difference satisfied preset thresholds. Agreement was additionally confirmed using an equivalent‐circle IoU of ~0.50, consistent with Dice/Jaccard criteria. Semantic segmentation was assessed with the Dice coefficient and IoU, and instance‐level detection with precision, recall, and F1 [20, 21].

### 2.7 Count‐Agreement Outcomes

Agreement between AI‐predicted and the reference vessel counts was evaluated using bias, mean absolute error (MAE), intraclass correlation coefficient (ICC [3,1]), and Bland–Altman analysis [22, 23]:

Bias=Pred−GT,  MAE=|Pred−GT|.

Bland–Altman limits of agreement were calculated as mean difference ±1.96 SD. All count comparisons were conducted at the ROI level. Proportional bias was assessed by linear regression of the difference (Pred‐GT) on the mean count, adjusting for ROI area. All tests were two‐sided, with *p* < 0.05 considered significant.

### 2.8 Sensitivity Analyses and Statistics

To assess robustness, three parameters were varied systematically:

Matching threshold (*α*): 0.1–1.0,

Minimum area threshold: 2–8 µm² (step = 1 µm²),

Non‐maximum suppression (NMS) factor: 0.3–0.7.

For NMS, overlapping predictions were merged when the centroid distance was ≤ 0.7 × min(*r*), retaining the object with the largest area. Sensitivity analyses confirmed that performance trends were consistent across all tested ranges. Because multiple ROIs came from the same slide, ROIs were treated as repeated measures (blocks). Global differences were tested with the Friedman test, and pairwise comparisons were assessed with the Wilcoxon signed‐rank test with Bonferroni correction when appropriate.

### 2.9 Software and Computational Environment

All analyses and figures were generated in Python 3.11.8 in an offline Jupyter environment to ensure repeatability. We used the following libraries: NumPy 1.24.0, pandas 1.5.3, SciPy 1.14.1, Matplotlib 3.6.3, and scikit‐learn 1.1.3. Spatial computations (e.g., polygon intersection) were handled using Shapely 1.7.1, and non‐maximum suppression was implemented with a custom centroid‐distance script. All analyses were performed offline without external web or cloud services.

## 3 Results

### 3.1 Segmentation and Detection

**Figure 3:**
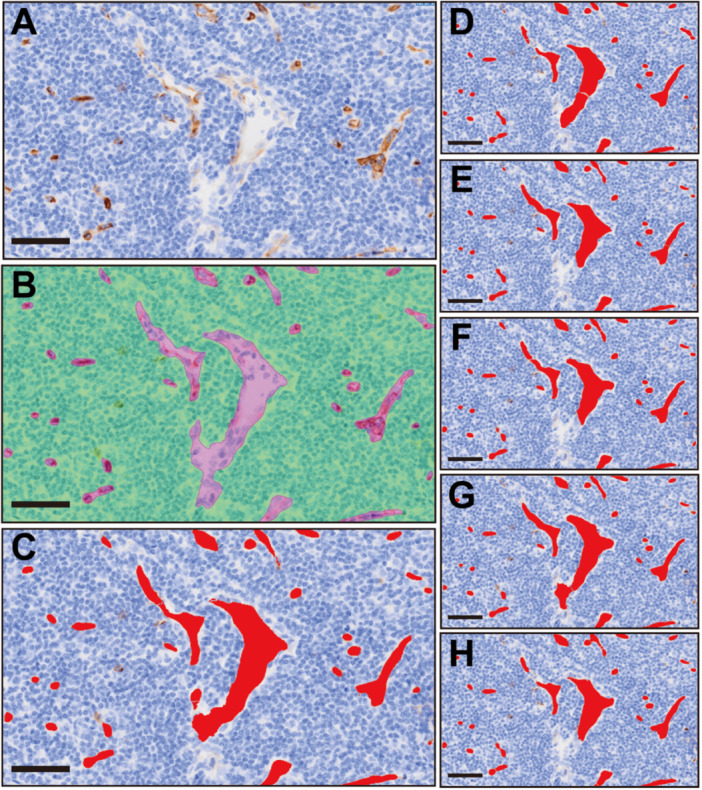
Qualitative comparison across training checkpoints. (A) Representative CD34‐immunostained field from the validation lymph node (L02): Weakly stained HEVs at the center with surrounding fine microvessels. (B) Ground truth annotations: AI‐assisted segmented reference vessel regions (magenta) and tissue/background mask (green). (C) Predictions of the best‐performing 3.0 M model (shown in red) before fixed post‐processing. (D–H) Object masks at training checkpoints 25 k, 2.0 M, 2.5 M, 3.5 M, and 4.0 M, respectively; the 3.0 M model shows the highest structural fidelity with minimal fragmentation. Scale bars = 50 µm. HEV, high‐endothelial venules; k, kilo; M, million.

We analyzed 13 lymph nodes—training set T01–T10 and validation set L01–L03—and summarized their area, perimeter, and vessel counts in Table 1. The model consistently detected CD34‐positive vasculature across diverse morphologies, including slender, elongated vessels and small, rounded capillaries. Unstained lumina lined by CD34‐positive endothelium were preserved as intravascular spaces within the segmented vessel objects, regardless of intraluminal blood cells (erythrocytes or leukocytes). Vessels with weak or focally negative CD34 staining were accurately segmented as single objects. The model produced some false positives, but their number remained limited even in regions with strong background staining, capsule‐adjacent tissue damage, or staining artifacts. These results suggest that the model maintained good specificity to true vascular morphology despite such challenging conditions. For large vessels, the model often failed to recognize a single continuous structure and instead produced short, fragmented segments. Most of these dot‐like fragments were removed by the post‐processing procedure, which merges adjacent sections and removes small debris. The predominant residual error was fragmented segmentation of large vessels, in which vessels were delineated as discontinuous outlines rather than a single continuous object (Figure 3).

### 3.2 Development Behavior (Pixel Level)

**Figure 4:**
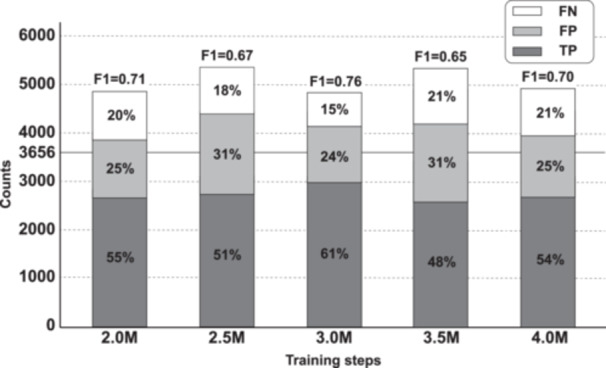
Balance of TP, FP, and FN across 2.0 M to 4.0 M, indicating the best precision–recall trade‐off at 3.0 M. ROI matching used centroid distance (*κ* = 0.5) and relative area error (*τ* = 0.30) with one‐to‐one Hungarian assignment. FN, false negative; FP, false positive; M, million; ROI, region of interest; TP, true positive.

On the held‐out validation set, Dice (0.913–0.928) and IoU (0.840–0.866) increased rapidly and plateaued near 3.0 M (Dice = 0.927, IoU = 0.872; Table 2). Beyond 3.5 M–4.0 M, both metrics declined slightly, consistent with mild overfitting reported for biomedical U‐Net training (Figure 4) [6]. We therefore set the operating point at 3.0 M for all downstream analyses (Table 2). The probability threshold was fixed at the 3.0 M checkpoint, where the validation Dice was highest, providing a balanced trade‐off between sensitivity to small vessels and avoidance of false detections.

**Table 2:** Pixel‐level performance across training iterations.

Training iterations	IoU	Dice
25 k	0.842	0.907
1.0 M	0.868	0.923
2.0 M	0.871	0.925
2.5 M	0.869	0.924
3.0 M	0.871	0.925
3.5 M	0.867	0.924
4.0 M	0.869	0.925

*Note:* IoU and Dice coefficient on the validation set across checkpoints, showing a plateau near 3.0 M that supports selection of the 3.0 M operating point. Abbreviations: IoU, intersection over Union; k, kilo; M, million.

### 3.3 Instance‐Level Performance

**Figure 5:**
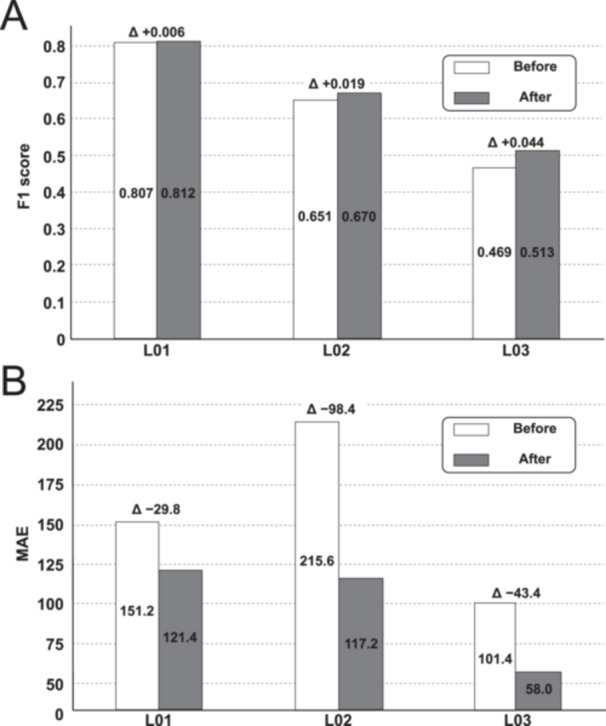
Effect of fixed post‐processing at the 3.0 million operating point. (A) F1‐score per ROI (L01–L03) before and after post‐processing. (B) MAE per ROI showing reduction after post‐processing. MAE, mean absolute error; ROI, region of interest.

At the instance level, the 3.0 M model achieved the highest F1 and the most balanced precision–recall across checkpoints from 2.0 M to 4.0 M. We did not analyze models below 2.0 M because Dice and IoU were too low on the validation set. Applying the fixed post‐processing procedure (ROI restriction → area ≥ 6 µm² → NMS 0.7) removed noise and duplicates while maintaining high recall. Predictions outside the ROI remained < 1%, confirming that the convex‐hull proxy ROI effectively captured the target area. Post‐processing reduced false positives and stabilized count accuracy (evaluated with MAE) without degrading F1 (Figure 5A,B). Mean F1 varied by only ±0.001 across NMS values of 0.3–0.7, indicating that vessel detection was robust to small parameter changes. MAE was lowest around 5–6 µm², so 6 µm² was adopted as the default threshold.

### 3.4 Per‐ROI Performance

Across lymph nodes L01–L03, instance‐level precision, recall, and F1 followed consistent trends, confirming stable inter‐ROI performance. Wilson 95% confidence intervals were narrow (±0.02–0.03), indicating well‐specified detection across slides.

### 3.5 Agreement at the ROI Level

At the ROI level, Bland–Altman analysis showed a small positive bias: on average, AI counts exceeded the reference by 105 vessels per ROI (≈+ 7.9%). The 95% limits of agreement are −110 to +320 vessels (≈−8.4% to +24.2%), with most data points inside this range. There is no significant proportional bias versus mean count (slope = 0.14, *p* = 0.23) or ROI area (slope = −2.6 × 10⁻⁵, *p* = 0.54). Given that all three ROIs are independent, this error range is acceptable for well‐specified vessel quantification and screening applications.

## 4 Discussion

Deep learning has been applied to segmentation and counting across numerous pathology tasks. Previous studies have analyzed vascular structures in glioma and on H&E‐stained slides [13, 14]. Unlike nuclei, cells, or glandular structures, blood vessels include complex branching and many with highly irregular outlines. Objects whose shapes deviate greatly from a circle are comparatively difficult to recognize as individual objects. So, instance‐level analyses of vessels have been limited. In this study, we made such analysis feasible by combining U‐Net–based morphological recognition with a standardized post‐processing procedure. Our procedure extends this concept to lymph‐node WSIs and emphasizes instance‐level agreement within a fixed post‐processing scheme. This design improves repeatability and comparability, aligning with previous work on HEV biology in inflammation and lymphoma [1, 2, 3, 4]. As digital pathology becomes increasingly integrated into diagnostics and research [5, 6, 7], standardized and transparent procedures such as ours will become essential.

This study presents a simple and transparent method for vessel counting in lymph‐node WSIs. Using one fixed operating point (3.0 M) and a single post‐processing procedure, the model yielded stable results with a small positive bias (Figure 4). Performance remains consistent across ROIs, and the overall design is easy to understand, replicate, and apply in practice.

The model accurately detected blood vessels with diverse morphologies and staining patterns, including slender, elongated vessels and small, circular microvessels. It correctly recognized HEVs with uneven staining and small capillaries, while avoiding misclassification of strongly stained but nonvascular regions such as capsule damage or disrupted tissue. Prior reports noted occasional misclassification of intravascular blood cells as tissue, but we did not observe this; adipocytes near the capsule were also not misclassified [17]. In some regions, the model marked vessels that appeared nearly CD34‐negative by inspection, suggesting reliance on hematoxylin‐visible structure—such as vessel walls and lumina—rather than stain intensity alone. For larger vessels, the model occasionally split one vessel into several objects, producing discontinuous, point‐like fragments at vessel borders. To prevent overcounting, post‐processing merged adjacent sections into single continuous objects. This approach effectively minimized fragment‐related artifacts and improved the consistency of instance‐level quantification across ROIs.

Case L03 shows lower F1. We interpret this mainly as a prevalence effect rather than a model failure. In these cases, Table 1 indicates a limited vascular load (fewer vessels and a smaller vascularized area), so even one FP or FN lowers F1 more than usual. Because of how F1 is computed, having few positives makes count‐based metrics more volatile and downward‐biased, even when pixelwise overlap remains steady. Based on the data tables, the lymph node size in L03 is small, and the number of blood vessels it contains is also limited. When the total number of vessels is small, the impact of false positives becomes more significant, so this likely has some influence. In addition, although the total vessel area in L03 is small, the perimeter is relatively long, suggesting that many of the vessels are thin. Such thin vessels are more sensitive to positional deviations, which are less tolerated under the current post‐processing conditions, and this may also be contributing to the result.

Accurate counting supports studies that use total vessel number or density as endpoints. A single operating point (3.0 M) keeps false‐negative risk low while it controls over‐detection. After filtering, the mean absolute error stays within a few percent at the ROI level, which is acceptable for many research tasks. F1 summarizes this balance. Agreement analysis (Bias, ICC, Bland–Altman) adds direct evidence that the counts are close enough for use in screening, triage, or pre‐quantification.

Good object detection opens use cases that simple pixel overlap cannot support. Examples include: diseases where perivascular lymphocyte clusters carry meaning (perivascular cuffs and related patterns); conditions with HEV expansion or remodeling, where we need per‐vessel masks to measure distribution, size, or shape; and spatial analysis around vessels (e.g., distance maps, neighborhood counts, region‐specific summaries such as capsule, cortex, and medulla). In these tasks, a reliable per‐object mask matters more than a small gain in pixel‐wise boundary score.

Each component addresses a common source of error. Removing predictions outside the capsule prevents false detections. Filtering out small fragments under 6 µm² yields the largest accuracy gain. Non‐maximum suppression (NMS = 0.7) removes duplicates while maintaining high recall. Pixel‐level Dice and IoU scores help select the model, whereas instance‐level and count‐agreement metrics confirm its practical utility. Together, these steps form a logical and standardized procedure. We fixed the operating point at 3.0 M to avoid test‐set overfitting. The post‐processing steps are straightforward and transparent, incorporating a standard non‐maximum suppression procedure. We report both instance‐level metrics (precision, recall, F1) and count‐agreement statistics (bias, MAE, ICC, Bland–Altman), following best practices for medical AI evaluation.

This single‐center, retrospective study has a limited number of lymph nodes, constraining generalizability. We did not assess domain shifts across scanners, laboratories, staining protocols, alternative endothelial markers, or extra‐nodal tissues. Our annotation rule merged vessel cross‐sections judged to be adjacent—particularly within germinal centers—into a single object; this choice affects counts and may introduce inter‐institutional variability. We used a single U‐Net‐based method with a fixed post‐processing procedure and did not compare alternative architectures or larger self‐configuring systems. In addition, due to hardware limitations, we did not assess training iterations beyond 4.0 M. Regarding evaluation, agreement was measured using instance‐level IoU on equivalent‐circle masks. Elongated vessels may be under‐scored; we partially mitigated this by combining centroid proximity with relative area matching, although the metric remains approximate. Because TruAI cannot export per‐ROI masks, some analyses used a convex‐hull ROI, which can include a small rim outside the true capsule.

In conclusion, we have developed an AI model that can detect blood vessels in lymph nodes WSIs with high accuracy. Future work will expand the validation set and include external validation across scanners and staining protocols. We also plan to evaluate performance in enlarged lymph nodes and to analyze detection by vessel size and anatomic region (capsule, cortex, medulla). Finally, we intend to release the post‐processing code to support consistent development in digital pathology.

## Author Contributions

All authors contributed to this article as follows: Satomi Omotani performed the research and wrote the manuscript; Natsumi Yonemoto designed and performed the research, and wrote the manuscript; Akifumi Muramoto analyzed the data, and wrote and revised the manuscript for important intellectual content; and Motohiro Kobayashi organized the research team, conceived of and designed the research, revised the manuscript. All authors have read and approved the final manuscript.

## Funding

The authors have nothing to report.

## Disclosure

The authors have nothing to report.

## Ethics Statement

This study was approved by the Research Ethics Committee of University of Fukui (reference number 20250013, approved on May 12, 2025) and we provide an opt‐out page (https://research.hosp.u-fukui.ac.jp/wp/wp-content/uploads/2025/05/20250013.pdf).

## Conflicts of Interest

The authors declare no conflicts of interest.

## Data Availability

The data that support the findings of this study are available from the corresponding author upon reasonable request. We can share anonymized per‐object tables and derived figures upon reasonable request.
